# Alcohol Consumption during Pregnancy: Analysis of Two Direct Metabolites of Ethanol in Meconium

**DOI:** 10.3390/ijms17030417

**Published:** 2016-03-22

**Authors:** Arantza Sanvisens, Neus Robert, José María Hernández, Paola Zuluaga, Magí Farré, Wifredo Coroleu, Montserrat Serra, Jordi Tor, Robert Muga

**Affiliations:** 1 Departments of Internal Medicine, Hospital Universitari Germans Trias i Pujol, Universitat Autònoma de Barcelona, 08916 Badalona, Spain; asanvisens.igtp.germanstrias@gencat.cat (A.S.); yzuluaga@igtp.cat (P.Z.); jordi.tor@uab.cat (J.T.); 2 Emergency Medicine, Hospital Universitari Germans Trias i Pujol, Universitat Autònoma de Barcelona, 08916 Badalona, Spain; bukatha@hotmail.com; 3 Proteomic and Metabolomic Unit, Fundació Institut d’Investigació Germans Trias i Pujol, 08916 Badalona, Spain; josemariahernandezp@yahoo.es; 4 Clinical Pharmacology, Hospital Universitari Germans Trias i Pujol, Universitat Autònoma de Barcelona, 08916 Badalona, Spain; mfarre.germanstrias@gencat.cat; 5 Paediatrics, Hospital Universitari Germans Trias i Pujol, Universitat Autònoma de Barcelona, 08916 Badalona, Spain; wcoroleu.germanstrias@gencat.cat; 6 Obstetrics and Gyneacology, Hospital Universitari Germans Trias i Pujol, Universitat Autònoma de Barcelona, 08916 Badalona, Spain; mserra.germanstrias@gencat.cat

**Keywords:** fetal ethanol exposure, meconium, ethyl glucuronide, ethyl sulfate

## Abstract

Alcohol consumption in young women is a widespread habit that may continue during pregnancy and induce alterations in the fetus. We aimed to characterize prevalence of alcohol consumption in parturient women and to assess fetal ethanol exposure in their newborns by analyzing two direct metabolites of ethanol in meconium. This is a cross-sectional study performed in September 2011 and March 2012 in a series of women admitted to an obstetric unit following childbirth. During admission, socio-demographic and substance use (alcohol, tobacco, cannabis, cocaine, and opiates) during pregnancy were assessed using a structured questionnaire and clinical charts. We also recorded the characteristics of pregnancy, childbirth, and neonates. The meconium analysis was performed by liquid chromatography—tandem mass spectrometry (LC-MS/MS) to detect the presence of ethyl glucuronide (EtG) and ethyl sulfate (EtS). Fifty-one parturient and 52 neonates were included and 48 meconium samples were suitable for EtG and EtS detection. The median age of women was 30 years (interquartile range (IQR): 26–34 years); EtG was present in all meconium samples and median concentration of EtG was 67.9 ng/g (IQR: 36.0–110.6 ng/g). With respect to EtS, it was undetectable (<0.01 ng/g) in the majority of samples (79.1%). Only three (6%) women reported alcohol consumption during pregnancy in face-to-face interviews. However, prevalence of fetal exposure to alcohol through the detection of EtG and EtS was 4.2% and 16.7%, respectively. Prevention of alcohol consumption during pregnancy and the detection of substance use with markers of fetal exposure are essential components of maternal and child health.

## 1. Introduction

Alcohol consumption is socially accepted in Western countries and the consequences of hazardous drinking are often minimized. In women of childbearing age, it may represent a serious health problem because of the risk of congenital malformations and neurodevelopmental alterations in newborns [1,2]. The extreme clinical effects of fetal exposure to alcohol were described decades ago [3]. Since then, the Fetal Alcohol Syndrome (FAS) has become a well-recognized clinical picture that is associated with high medical consequences and social costs. In the United States, FAS is considered the primary cause of preventable birth defects [4,5] and the most serious consequence of drinking alcohol during pregnancy, with the exception of intrauterine fetal death. Clinically, FAS is characterized by low birth weight, a distinctive phenotype with central nervous system alterations and behavioral deficiencies during development. The incidence of FAS is 0.5–1.5 per 1000 births per year [6], although the range of clinical effects induced by alcohol consumption during pregnancy is much greater and includes Fetal Alcohol Spectrum Disorder (FASD). The incidence of FASD is approximately 1% of live births [6,7].

In recent years, it has become clear that adolescent and young women from Western countries are drinking alcohol at a similar rate to men. In Spain, approximately 70% of young women have consumed alcohol in the last year, and 50% have consumed alcohol in the last month [8].

According to a study in the US, 60% of women of childbearing age consume alcohol occasionally, and 13% exhibit hazardous drinking, with alcohol intake >7 Standard Drink Units (SDU) per week [9]. In addition, in the US, a survey finds that approximately one in eight pregnant women report alcohol use [10].

The early diagnosis of alcohol consumption in pregnant women is hampered by the absence of laboratory markers of continued exposure. During the last decade, efforts have been made to identify biological markers of exposure during gestation. Clinical interviews and history taking in pregnant women usually underdiagnose alcohol exposure [11]. The majority of studies on markers of alcohol consumption during pregnancy have focused on ethanol metabolites derived from non-oxidative metabolism, such as fatty acid ethyl esters (FAEEs) [12,13,14], and in the enzymatic conjugation of ethanol with glucuronide and sulfate [15,16,17,18]. Within the last few years, ethyl glucuronide (EtG) and ethyl sulfate (EtS) have been demonstrated to be markers of fetal alcohol exposure with good sensitivity and specificity [19]. Meconium, the first fecal matter of the newborn, is a useful substrate for analysis because it reveals all fetal metabolism from the 12th week of pregnancy onward [20,21]. However, current research suggests that FAEEs may not be adequate markers of alcohol exposure due to degradation susceptibility arising from environmental conditions [22,23]; in addition, few studies have simultaneously analyzed both EtG and EtS in meconium samples [24,25,26]. In addition, the method of choice for detecting and quantifying direct ethanol metabolites in the meconium is liquid chromatography-tandem mass spectrometry (LC-MS/MS) [27], a technique that allows the separation and quantification of different component elements and analysis of their molecular mass [28].

We hypothesized that parturient women barely self-report alcohol consumption and that a proportion of asymptomatic newborns have been exposed to ethanol. In this sense, we aimed to analyze prevalence of alcohol consumption in mothers and fetal exposure to ethanol in a series of parturient women and their newborns following childbirth.

## 2. Results

### 2.1. Characteristics of the Study Population

The study population consisted of 51 women and 52 meconium samples (one of the women gave birth to twins). The median age of the women was 30 years (IQR: 26–34 years). The obstetrics data (Table S1), pregnancy outcomes (Table S2), and characteristics of newborns (Table S3) are shown in Appendix A.

According to the questionnaire and clinical charts, overall prevalence of any substance (tobacco, alcohol, cannabis, cocaine, opiates) use during pregnancy was 41.2% (21/51) and tobacco was the substance most frequently used (86.7%). Twelve (57%) out of 21 women were substance users but only 3 out of 12 self-reported alcohol consumption during pregnancy. In 43% (9/21), history of substance use during pregnancy was obtained by reviewing clinical charts.

### 2.2. EtG, EtS Determination and Substance Use

**Figure 1:**
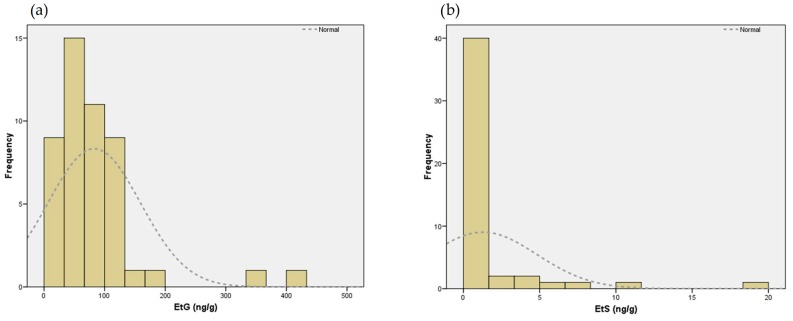
Distribution of two direct metabolites of ethanol in 48 meconium samples: (**a**) EtG; and (**b**) EtS.

Of the 52 meconium samples, 48 were suitable for the analysis of EtG and EtS. EtG was present in all meconium samples and median concentration of EtG was 67.9 ng/g (IQR: 36.0–110.6 ng/g). Figure 1a shows the distribution of EtG concentration in meconium. According to the cut-off used (274 ng/g), two meconium samples tested positive for ethanol despite clinical records, and interview on alcohol consumption in the respective mothers was negative.

With respect to EtS, it was undetectable (<0.01 ng/g) in the majority of meconium samples (79.1%), and 16.7% tested positive (cut-off: 1.51 ng/g); however, the interview and clinical records for the respective mothers was negative.

Figure 1b shows the distribution of EtG and EtS concentration in meconium samples.

**Figure 2:**
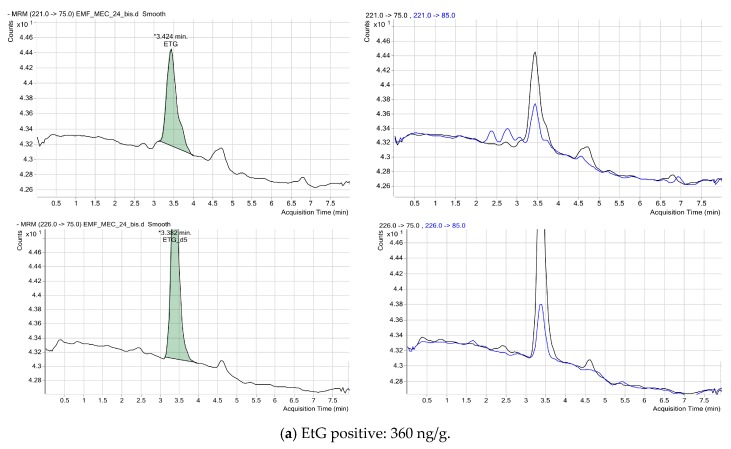

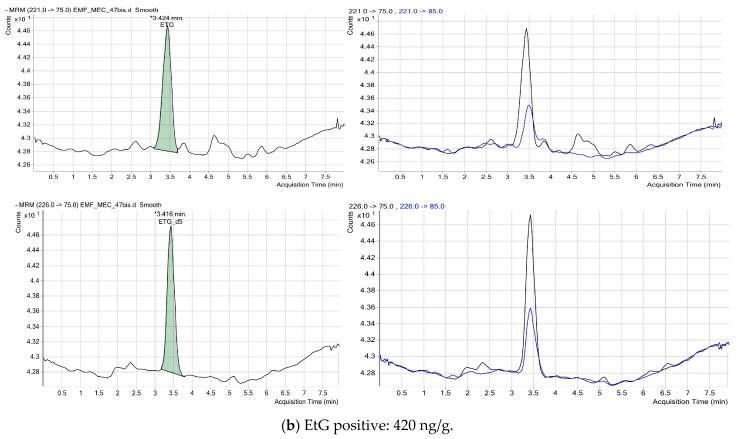
Chromatogram in two meconium samples testing positive for EtG. The retention time of substance ranged from 3 to 3.5 min depending on the column life span. The black line depicts the quantitative transitions and the blue line depicts the qualitative transitions. The green shade represents the quantification of EtG using the calibration curves. The “multiple reaction monitoring” (MRM) program takes into account the following transitions: EtG quantifier 221 > 75 and qualifier 221 > 85, internal standard (EtG-d5) quantifier 226 > 75 and qualifier 226 > 85. (**a**) sample EMF_MEC_24 with a positive concentration of 360 ng/g; (**b**) sample EMF_MEC_47 with a positive concentration of 420 ng/g.

**Figure 3:**
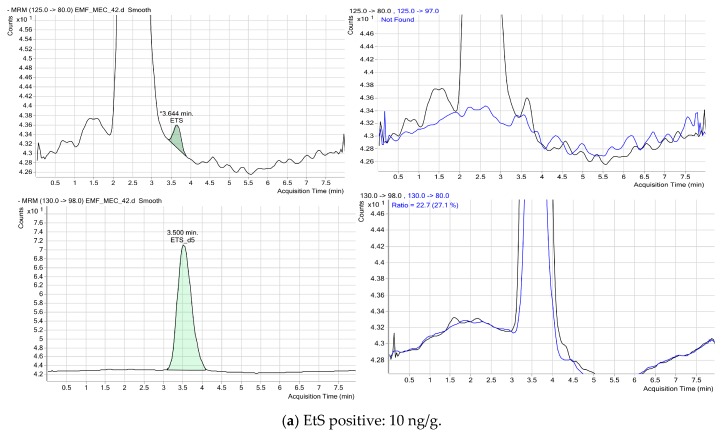

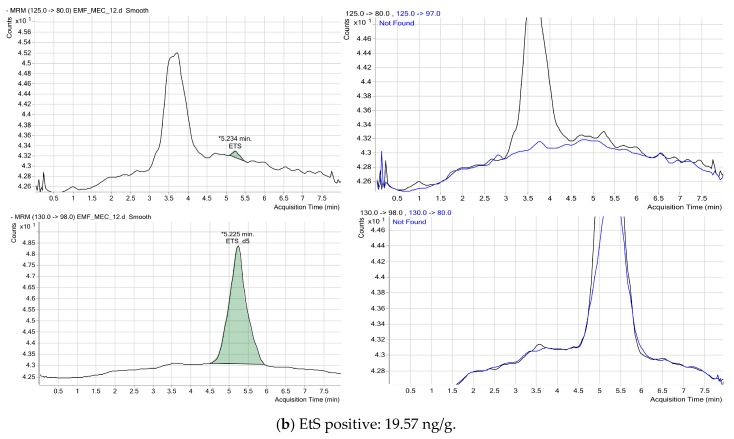
Chromatogram in two meconium samples testing positive for EtS. The retention time of substance ranged from 4.5 to 5.5 min depending on the column life span. The black line depicts the quantitative transitions and the blue line depicts the qualitative transitions. The green shade represents the quantification of EtS using the calibration curves. The “multiple reaction monitoring” (MRM) program takes into account the following transitions: EtS quantifier 125 > 80 and qualifier 125 > 97, and internal standard (EtS-d5) quantifier 130 > 98 and qualifier 130 > 80. (**a**) sample EMF_MEC_42 with a positive concentration of 10 ng/g; (**b**) sample EMF_MEC_12 with a positive concentration of 19.57 ng/g.

Figure 2 shows the chromatograms for two positive cases for EtG, and Figure 3 shows the chromatograms for two positive cases for EtS. As shown in the figures, the qualifier trace was disturbed by overlapping matrix peaks, and there were no disturbing signals from matrix constituents at EtG and EtS concentrations. No additional peaks due to endogenous substances that could have interfered with the detection of metabolites were observed.

The detection of positive levels of EtG or EtS in the meconium samples was not associated with age of mothers, TPAL (number of term births (T)/number of preterm births (P)/number of abortions (A)/number of living children (L)), gestational age, compliance of women with antenatal visits, weight gain during pregnancy, or weight of newborns.

Table 1 summarizes the prevalence of substance use with regard to the information obtained via structured questionnaires and the detection of direct ethanol metabolites in the meconium samples.

**Figure 4:**
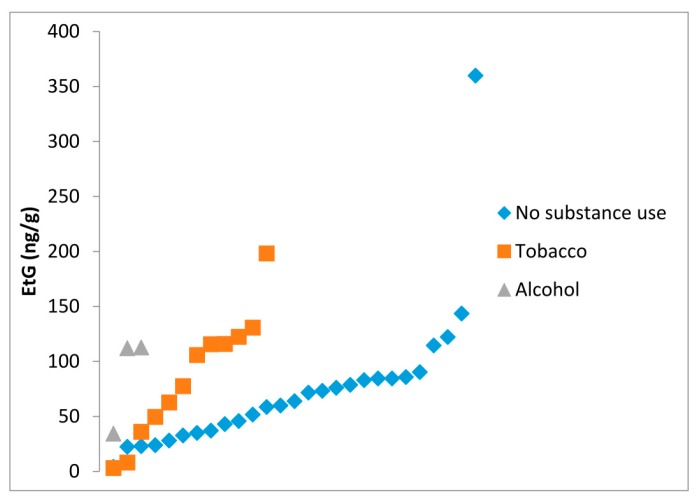
EtG values (ng/g) in meconium samples of newborns exposed and non-exposed to tobacco and alcohol during pregnancy.

Although not statistically significant, EtG values in the meconium samples were higher in the 21 parturients with substance use: 64 ng/g (IQR: 35–84 ng/g) in those without substance use, 91 ng/g (IQR: 39.5–120.9 ng/g) in tobacco smokers, and 112 ng/g (IQR: 34.4–112.8 ng/g) in those drinking alcohol (*p* = 0.375). Figure 4 shows all the EtG values according to substance use during pregnancy.

## 3. Discussion

Ethyl glucuronide (EtG) and ethyl sulfate (EtS) have been demonstrated to be good markers of fetal alcohol exposure [19]. EtG is formed by the ethanol conjugation with glucuronic acid and it is formed almost entirely in the liver; EtS is formed by the transfer of a sulfuric group from 3’-phosphoadenosine- phosphosulfate to ethanol in the mitochondria. The results obtained in this study confirm that these metabolites can be determined in meconium by methods that have been widely validated [28,29].

EtG and EtS are polar, water soluble and stable compounds; LC-MS/MS is the method of choice for the detection of direct metabolites of ethanol [27,28]. However, a new method for the detection of EtG–hydrophilic interaction liquid chromatography-tandem mass spectrometry (HILIC-MS/MS)—has been recently introduced [30]. In any case, both LC-MS/MS and HILIC-MS/MS, unlike others such as gas chromatography/flame ionization (GC/FID) and gas chromatography/mass spectrometry (GC/MS), minimize manual labor by eliminating the solid-phase extraction [30,31,32].

In this study, the distribution of EtS in meconium samples was similar to that observed in two Mediterranean cohorts [12,19]; however, in our study median EtG (64 ng/g) was higher than observed in newborns from Reggio-Emilia from Italy (15.6 ng/g) but it was lower than reported in another study in Barcelona, Spain (101.5 ng/g) [19]. Interestingly, few studies have simultaneously analyzed both EtG and EtS in meconium samples [24,25,26].

Studies of markers of fetal alcohol exposure during pregnancy have shown that EtG and EtS have similar diagnostic sensitivity and specificity [15,16,17,18]. In the present study, we did not evaluate EtG and EtS as diagnostic tests, but results indicate that, irrespective of the substance used during pregnancy, EtG levels were higher in meconium samples of newborns whose mothers reported substance use compared with pregnant women without substance use. In fact, EtG dose-dependently detects ethanol consumption [18], and a well-known association exists between tobacco smoke and alcohol consumption [33,34,35]. In the present study, higher levels of EtG in newborns of mothers that were tobacco smokers could indicate occasional alcohol consumption during pregnancy. A study from Ireland shows that smoking during pregnancy is associated with alcohol consumption and/or other substance use [36]. More predictors of alcohol drinking during pregnancy include prior history of consumption and the antecedent of binge drinking [37].

In the present study, only 6% of parturient women report drinking alcohol during pregnancy and 23% report any substance use including tobacco, cannabis, cocaine, opiates, or alcohol. However, when the face-to-face interview is complemented by the review of clinical charts, the prevalence of any substance use during pregnancy is twice as high. Differences in prevalence of substance use during pregnancy may be related to the minimization or denial of risk behavior by the women after childbirth [38,39]. Furthermore, some studies have demonstrated the low sensitivity of questionnaires regarding substance use in pregnant women [11,39]. One recent study on the use of illicit drugs in Spain showed that only 2% of mothers self reported substance use, whereas the prevalence of consumption detected by biological markers was 16% [40]. In this study, the results on substance use from questionnaires did not correlate with those obtained with markers of fetal exposure, as reported previously [19,40]. A recent meta-analysis indicates that prevalence of prenatal alcohol exposure as measured by FAEEs in meconium was four times more frequent than prevalence measured by self-reports [41]. In this study only 6% of the women report alcohol drinking during pregnancy whereas prevalence of fetal alcohol exposure detected by EtG and EtS in meconium was 4.2% and 16.7%, respectively.

The prevalence of fetal alcohol exposure in this study, regardless of whether it was detected through the review of the clinical records or through the detection of metabolites in the meconium samples, was lower than observed in previous studies [42,43,44]. This funding may be related to the fact that in our study 29% of the women originated from countries where alcohol drinking is not socially accepted which could favor a lower prevalence of alcohol consumption in pregnant women. Another explanation would be related to the fact that the meconium samples in the present study were obtained from healthy newborns excluding those cases admitted to a neonatal care unit.

The present study has limitations that should be mentioned. First, the assessment of substance use in a small number of women and newborns limits the generalization of the findings; Second, results are based on asymptomatic neonates with normal birth weights and Apgar score, thus indicating a possible selection bias toward healthy newborns. Low birth weight is known to be one of the clinical manifestations of FASD, and newborns of mothers with substance abuse usually need to be admitted to intensive neonatal care units; Third, parturients with significant substance use disorders may have declined to participate in the study. In fact, of the 62 women who were invited to participate, 17.7% declined. Finally, the cut-off for EtG and EtS used in this study were suggested by previous studies taking into account the cut-off used in the initial evaluation of FAEEs [29].

## 4. Materials and Methods

Cross-sectional study conducted in September 2011 and March 2012 in a series of parturient women who were admitted to the Obstetric Unit at Hospital Universitari Germans Trias i Pujol in Badalona, Spain, following childbirth and, in the meconium samples from their newborns.

Sixty-two women were informed of the objective of the study; of them, 51 agreed and 11 declined. The study was approved by the Ethics Committee of the Hospital Universitari Germans Trias i Pujol. The procedures complied with ethical norms for medical investigations and the principles of good practice established by the Declaration of Helsinki.

### 4.1. Collection of Clinical Data and Biological Samples

A structured questionnaire was administered by a physician to obtain socio-demographic, and substance use characteristics. This information was complemented by a systematic review of charts, including the pregnancy characteristics and childbirth records.

We collected the following data from participants: age, anthropometric measurements at the beginning and the end of pregnancy, history of medical co-morbidity (e.g., hypertension, diabetes mellitus), the obstetric record via TPAL algorithm, the pharmacological treatment during pregnancy, biochemical and hematologic parameters from the first trimester of pregnancy, and the degree of compliance with antenatal visits. With respect to substance use, the administered questionnaire included the type of substance use including tobacco, alcohol, cannabis, cocaine, and opiate consumption during pregnancy.

Risk pregnancy was categorized in four levels (no risk, moderate, high, and very high) according to age of women, substance use, medical co-morbidity, obstetric history and socioeconomic status [45].

To characterize pregnancy outcomes, the following data were collected: week of gestation at birth, type of birth, birth weight, Apgar test score and immediate birth complications (<48 h).

### 4.2. Processing and Analysis of Meconium Samples

Meconium samples were refrigerated at 4 °C and frozen at −80 °C. Aliquots were analyzed for EtG and EtG using LC-MS/MS according to a validated method [29].

Sample preparation: 10 to 15 mg of the meconium were diluted with internal standards (EtG-d5 and EtS-d5; Medichem LGC Standards, Wesel, Germany). To homogenize and facilitate separation, the samples were diluted with 450 µL of water and 500 µL methanol and then sonicated. The samples were subsequently centrifuged at 16,100 rcf for 30 min and the supernatant was passed by a 3 kDa filter (Amicon Millipore, Cork, Ireland) and centrifuged at 16,100 rcf for 45 min.

Instrument analysis: Analysis of EtG and EtS was performed by LC-MS/MS using an Agilent 1260 Infinity/6410BA equipment (Agilent Technologies, Palo Alto, CA, USA), which included a Thermo Hypercarb Column liquid chromatography column (100 mm × 2.1 mm × 5 µm) with a Javelin Guard Hypercarb (Thermo Fisher Scientific, Waltham, MA, USA). Sixty microliters of the supernatant was injected at an initial rate of 0.3 mL/min (water:acetonitrile:formic acid, 0.1% *v*/*v*) at 45 °C. Table 2 shows the chromatograph programming.

Tandem mass spectrometry was performed in negative electrospray ionization (ESI) mode with a capillary voltage of 4000 V, source temperature of 350 °C, curtain gas at 13 L/min, and nebulizer pressure of 60 psi. The MRM program takes into account the following transitions: EtG quantifier 221 > 75 and qualifier 221 > 85, internal standard (EtG-d5) quantifier 226 > 75 and qualifier 226 > 85, EtS quantifier 125 > 80 and qualifier 125 > 97, and internal standard (EtS-d5) quantifier 130 > 98 and qualifier 130 > 80. A summary of MRM is shown in Table 3.

Calibration: a 7-point calibration curve for EtG and EtS was prepared by adding appropriate volumes of working calibrators and 50 μL of the internal standard (EtG-d5 and EtS-d5) solutions to 0.2 μg/mL of EtG/EtS-free meconium. The calibration curves were linear, with slopes of 0.5–0.7 for EtG and 0.11–0.2 for EtS, *r*^2^ > 0.99. Two selected calibration curves are shown in Appendix B, Figure S1. The sample concentrations were calculated according to the area under the curve of the sample divided by the area under the curve of the internal standards, extrapolating from the calibration curve.

### 4.3. Statistical Analysis

Continuous variables are presented as the median and IQR, and categorical variables are presented as relative frequencies. The cut-off to determine a positive result (exposure to ethanol) was 274 ng/g for EtG and 1.51 ng/g for EtS [29].

*χ*^2^ tests, Fisher F tests, Student’s *t*-test, and Mann–Whitney U tests were used to detect significant differences. Values of *p* < 0.05 were considered statistically significant. The statistical analysis was performed using SPSS 15.0 software (SPSS, Chicago, IL, USA).

## 5. Conclusions

In conclusion, this study provides data of two direct markers of fetal alcohol exposure that confirm a promising strategy to monitoring substance use in maternal and childcare. The current difficulties in the early diagnosis of alcohol consumption and substance use during pregnancy makes it necessary to improve the assessment of fetal exposure using efficient techniques.

## Figures and Tables

**Table 1:** Prevalence of substance use during pregnancy according to clinical data of parturient women and markers of fetal alcohol exposure.

	Clinical Information of Parturient Women		Markers of Fetal Alcohol Exposure	
Substance	Structured Questionnaire *n* (%)	Structured Questionnaire and Chart Review *n* (%)	EtG *n* (%)	EtS *n* (%)
Alcohol	3 (6)	3 (6)	2 (4.2)	8 (16.7)
Tobacco	7 (12)	17 (33.3)	NA	NA
Cannabis, Cocaine and/or Opiates	2 (3.9)	3 (6)	NA	NA

NA: not available.

**Table 2:** Chromatograph programming.

Time (min)	Flow (mL/min)	Phase A: CH_3_CN 0.1%, Formic Acid %	Phase B: H_2_O 0.1%, Formic Acid %
0	0.3	8	92
1	0.3	8	92
4	0.4	0	100
6	0.4	0	100
6.5	0.3	8	92
8	0.3	8	92

**Table 3:** Summaries of multiple reaction monitoring (MRM) for 8 channels.

Component	Internal Standard	Precursor	Fragment	Dwell Time (msec)	Fragmentor (Volts)	Collision Energy (ev)
EtG_d5	IS	226	85	250	100	15
EtG_d5	IS	226	75	250	100	15
EtG	‒	221	85	250	100	15
EtG	‒	221	75	250	100	15
EtS_d5	IS	130	98	250	100	15
EtS_d5	IS	130	80	250	100	15
EtS	‒	125	97	250	100	15
EtS	‒	125	80	250	100	15

## Supplementary Materials

Supplementary materials can be found at http://www.mdpi.com/1422-0067/17/3/417/s1.

## Abbreviations

EtG: ethyl glucuronide
EtS: ethyl sulfate
FAEE: fatty acid ethyl ester
FAS: Fetal Alcohol Syndrome
FASD: Fetal Alcohol Spectrum Disorder
IQR: interquartile range
LC-MS/MS: liquid chromatography—tandem mass spectrometry
NA: not available
TPAL: number of term births [T]/number of preterm births [P]/number of abortions [A]/number of living children [L]
