# Supplementary material for: Alcohol Consumption during Pregnancy: Analysis of Two Direct Metabolites of Ethanol in Meconium
Source: Int J Mol Sci. 2016 Mar 22;17(3):417. doi: 10.3390/ijms17030417 (PMC4813268; doi:10.3390/ijms17030417)
Supplement: Supplementary file 1 [file ijms-17-00417-s001.pdf]

Supplementary Materials: Alcohol Consumption during Pregnancy: Analysis of Two Direct Metabolites of Ethanol in Meconium

Arantza Sanvisens, Neus Robert, José María Hernández, Paola Zuluaga, Magí Farré, Wifredo Coroleu, Montserrat Serra, Jordi Tor and Robert Muga

Appendix A:

Table S1. Obstetric characteristics of 51 parturient women admitted to the Obstetric Unit in September 2011 and March 2012.

Characteristics	*N* = 51 *n* (%)
**Age (Median (IQR))**	30 (26–34)
**Obstetric History (TPAL)**	
Number of Term Births (T)	
0	22 (43.1)
1	23 (45.1)
2	4 (7.9)
3	2 (3.9)
Number of Preterm Births (P)	
0	51 (100.0)
Number of Abortions (A)	
0	36 (70.6)
1	9 (17.6)
2	4 (7.9)
3	2 (3.9)
Number of Living Children (L)	
0	22 (43.1)
1	23 (45.1)
2	4 (7.9)
3	2 (3.9)
**Comorbidities**	25 (49.0)
**Regular pharmacological treatment**	16 (31.4)

TPAL: number of term births (T)/number of preterm births (P)/number of abortions (A)/number of living children (L).

Table S2. Pregnancy outcomes of 51 parturient women admitted to the Obstetric Unit in September 2011 and March 2012.

Pregnancy Variables	*N* = 51 *n* (%)
**Risk Pregnancy Level (*n* = 44)**	
No risk	10 (22.7)
Moderate	18 (40.9)
High	16 (36.4)
Very high	0 (0)
**Completed Clinical Visits (*n* = 41)**	
80%–100%	31 (75.6)
51%–79%	5 (12.2)
≤50%	5 (12.2)

Table S2. *Cont.*

Pregnancy Variables	N = 51 n (%)
**Analysis of First Trimester (n = 42), median (IQR)**	
Haemoglobin (×10⁹/L)	11.7 (11.4–12.5)
Platelets (×10⁹/L)	217 (190–256)
Ferritin (ng/mL)	16 (11–28.9)
**Ultrasound of First Trimester (n = 39)**	
Normal	35 (89.7)
Altered	4 (10.3)
**Weight Increase During Pregnancy (n = 27), median (IQR)**	11.3 (8.4–16.3)
**Type of Birth (n = 52 *)**	
Eutocic	29 (55.7)
Dystocia (forceps, vacuum, other)	9 (17.3)
Caesarean (elective or urgent)	14 (27.0)
Gestational age in weeks, median (IQR)	40 (38.9–41)

* One of the women gave birth to twins.

Table S3. Characteristics of 52 newborns.

Characteristics	N = 52 n (%)
**Apgar Test Score at Birth**	
8	3 (5.8)
9	47 (90.4)
10	2 (3.8)
**Apgar Test Score at 5 min**	
10	52 (100.0)
**Immediate Birth Complications**	
No	50 (96.2)
Yes	2 (3.8)
**Birth weight (g), median (IQR)**	3240 (3050–3675)

Appendix B:

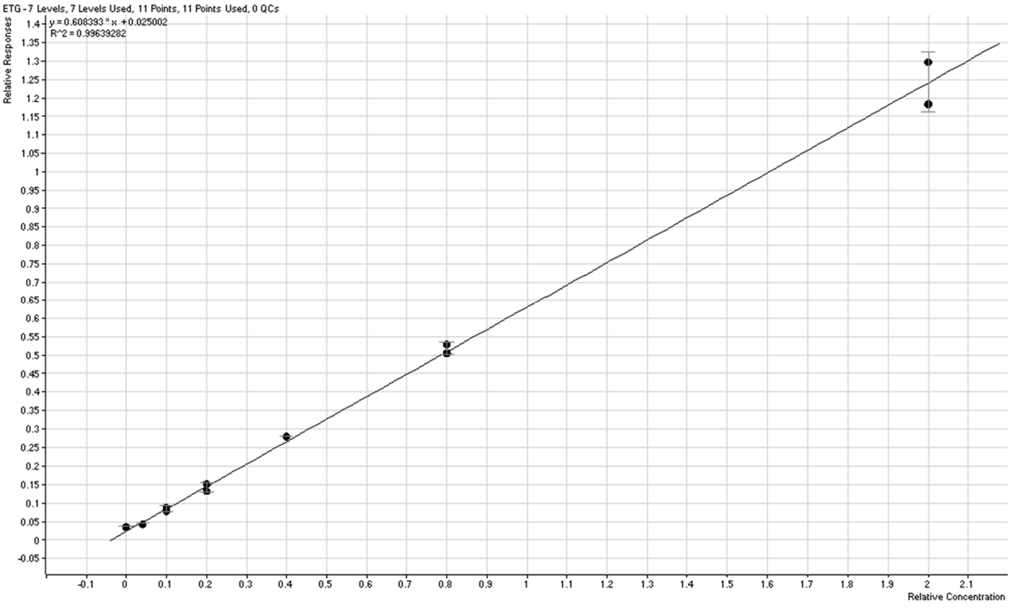

(**a**) EtG

Figure S1. *Cont.*

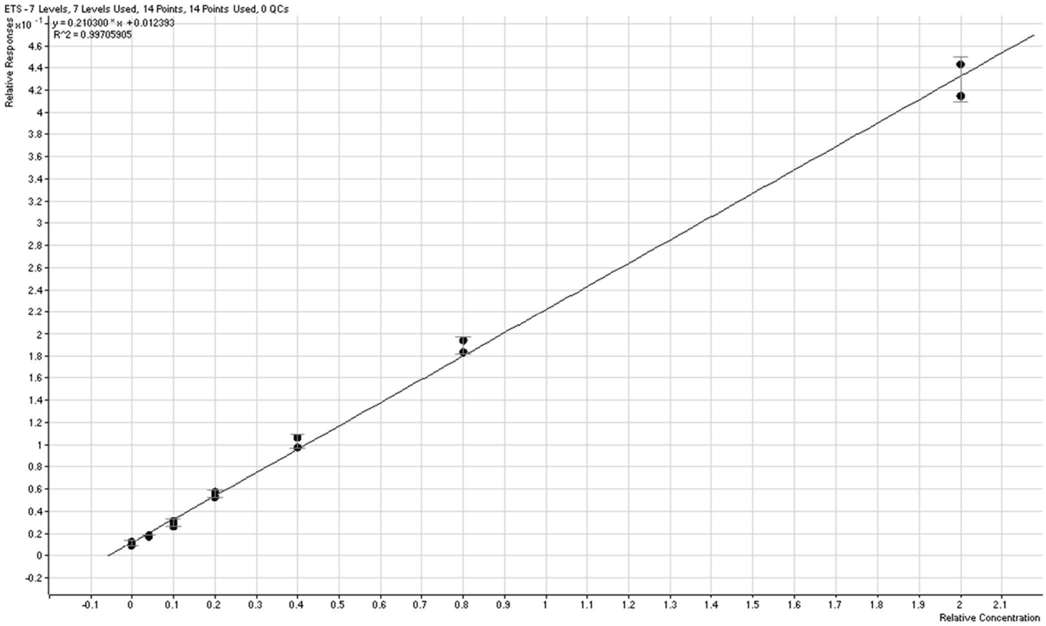

(**b**) **EtS**

Figure S1. Selected linear calibration curves for EtG (**a**) and EtS (**b**).
